# Thrifty wide-context models of B cell receptor somatic hypermutation

**DOI:** 10.1101/2024.11.26.625407

**Published:** 2024-12-01

**Authors:** Kevin Sung, Mackenzie M. Johnson, Will Dumm, Noah Simon, Hugh Haddox, Julia Fukuyama, Frederick A Matsen

## Abstract

Somatic hypermutation (SHM) is the diversity-generating process in antibody affinity maturation. Probabilistic models of SHM are needed for analyzing rare mutations, for understanding the selective forces guiding affinity maturation, and for understanding the underlying biochemical process. High throughput data offers the potential to develop and fit models of SHM on relevant data sets. In this paper we model SHM using modern frameworks. We are motivated by recent work suggesting the importance of a wider context for SHM, however, assigning an independent rate to each k-mer leads to an exponential proliferation of parameters. Thus, using convolutions on 3-mer embeddings, we develop “thrifty” models of SHM that have fewer free parameters than a 5-mer model and yet have a significantly wider context. These offer a slight performance improvement over a 5-mer model. We also find that a per-site effect is not necessary to explain SHM patterns given nucleotide context. Also, the two current methods for fitting an SHM model — on out-of-frame sequence data and on synonymous mutations — produce significantly different results, and augmenting out-of-frame data with synonymous mutations does not aid out-of-sample performance.

## Full Text Availability

The license terms selected by the author(s) for this preprint version do not permit archiving in PMC. The full text is available from the preprint server.

